# Incorporating citizen science into IUCN Red List assessments

**DOI:** 10.1111/cobi.14329

**Published:** 2024-08-27

**Authors:** Rachael Gallagher, Erin Roger, Jasmin Packer, Cameron Slatyer, Jodi Rowley, Will Cornwell, Emilie Ens, Sarah Legge, Colin Simpfendorfer, Ruby Stephens, Thomas Mesaglio

**Affiliations:** ^1^ Hawkesbury Institute for the Environment Western Sydney University Penrith New South Wales Australia; ^2^ Commonwealth Scientific and Industrial Research Organisation (CSIRO) Atlas of Living Australia Canberra Australian Capital Territory Australia; ^3^ Environment Institute The University of Adelaide Adelaide South Australia Australia; ^4^ School of Biological Sciences The University of Adelaide Adelaide South Australia Australia; ^5^ Centre for Ecosystem Science, School of Biological, Earth and Environmental Sciences (BEES) University of New South Wales Sydney New South Wales Australia; ^6^ Australian Museum Research Institute Australian Museum Sydney New South Wales Australia; ^7^ School of Natural Sciences Macquarie University Sydney New South Wales Australia; ^8^ Research Institute of Environment and Livelihoods Charles Darwin University Casuarina Northern Territory Australia; ^9^ Fenner School Environment and Society The Australian National University Acton Australian Capital Territory Australia; ^10^ College of Science and Engineering James Cook University Townsville Queensland Australia

**Keywords:** citizen science, conservation assessment, IUCN Red List, monitoring, observations, threat assessment, ciencia ciudadana, evaluación de amenazas, evaluación de la conservación, Lista Roja de la UICN, monitoreo, observaciones

## Abstract

Many citizen scientists are highly motivated to help address the current extinction crisis. Their work is making valuable contributions to protecting species by raising awareness, identifying species occurrences, assessing population trends, and informing direct management actions, such as captive breeding. However, clear guidance is lacking about how to use existing citizen science data sets and how to design effective citizen science programs that directly inform extinction risk assessments and resulting conservation actions based on the International Union for Conservation of Nature (IUCN) Red List criteria. This may be because of a mismatch between what citizen science can deliver to address extinction risk and the reality of what is needed to inform threatened species listing based on IUCN criteria. To overcome this problem, we examined each IUCN Red List criterion (A–E) relative to the five major types of citizen science outputs relevant to IUCN assessments (occurrence data, presence–absence observations, structured surveys, physical samples, and narratives) to recommend which outputs are most suited to use when applying the IUCN extinction risk assessment process. We explored real‐world examples of citizen science projects on amphibians and fungi that have delivered valuable data and knowledge for IUCN assessments. We found that although occurrence data are routinely used in the assessment process, simply adding more observations of occurrence from citizen science information may not be as valuable as inclusion of more nuanced data types, such as presence–absence data or information on threats from structured surveys. We then explored the characteristics of citizen science projects that have already delivered valuable data to support assessments. These projects were led by recognized experts who champion and validate citizen science data, thereby giving greater confidence in its accuracy. We urge increased recognition of the value of citizen science data within the assessment process.

## Abstract (es)

Integración de la ciencia ciudadana a las evaluaciones de la Lista Roja de la UICN

**Resumen:**

Existe mucha motivación entre los ciudadanos científicos para ayudar a enfrentar la actual crisis de extinción. Sus contribuciones son valiosas para la protección de las especies mediante la concientización, la detección de especies, la evaluación de las tendencias poblacionales y la información sobre acciones directas de gestión, como la cría en cautiverio. Sin embargo, faltan directrices claras sobre cómo utilizar los conjuntos de datos de ciencia ciudadana existentes y cómo diseñar programas de ciencia ciudadana eficaces que informen directamente las evaluaciones del riesgo de extinción y las acciones de conservación resultantes basadas en los criterios de la Lista Roja de la Unión Internacional para la Conservación de la Naturaleza (UICN). Esto puede deberse a un desajuste entre lo que la ciencia ciudadana puede aportar para abordar el riesgo de extinción y la realidad de lo que se necesita para fundamentar la inclusión de especies amenazadas en las listas según los criterios de la UICN. Para superar este obstáculo, analizamos cada criterio de la Lista Roja de la UICN (A‐E) en relación a los cinco tipos principales de resultados de la ciencia ciudadana relevantes para las evaluaciones de la UICN (datos de presencia, observaciones de presencia‐ausencia, encuestas estructuradas, muestras físicas y narraciones) para recomendar cuáles resultados son los más adecuados para la evaluación del riesgo de extinción de la UICN. Exploramos ejemplos reales de proyectos de ciencia ciudadana sobre anfibios y hongos que han aportado datos y conocimientos valiosos para las evaluaciones de la UICN. Descubrimos que, aunque los datos de presencia se utilizan de forma rutinaria en el proceso de evaluación, la adición de más observaciones de presencia a partir de información de ciencia ciudadana puede no ser tan valioso como la inclusión de tipos de datos más matizados, como datos de presencia‐ausencia o información sobre amenazas a partir de encuestas estructuradas. Después analizamos las características de los proyectos de ciencia ciudadana que ya han aportado datos valiosos en apoyo de las evaluaciones. Estos proyectos fueron dirigidos por expertos reconocidos que defienden y validan los datos de la ciencia ciudadana, lo que da mayor confianza en su exactitud. Instamos a un mayor reconocimiento del valor de los datos de la ciencia ciudadana en el proceso de evaluación.

## INTRODUCTION

**FIGURE 1:**
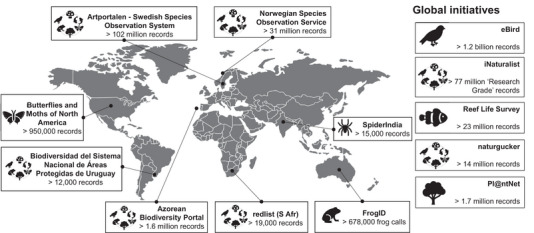
Examples of citizen science projects yielding valuable data for International Union for Conservation of Nature (IUCN) assessments and threatened species management. All data are available via Global Biodiversity Information Facility (GBIF).

Citizen science for biodiversity monitoring has arrived (Chandler et al., 2017; Fontaine et al., 2022). Recent initiatives demonstrate that, collectively, citizen scientists are capturing unprecedented volumes of data (Figure 1) (Aristeidou et al., 2021; Perry et al., 2022; Peter et al., 2019; Unger et al., 2021). In many cases, citizen scientists are inspired to halt extinctions and protect threatened species (Soroye et al., 2022; Steven et al., 2019). One of the most direct ways citizen scientists can effect change is through contributing data that can be harnessed by threat assessment processes, such as those of the International Union for Conservation of Nature (IUCN). Although this is not the primary way citizen scientists can support biodiversity, their data can directly inform the process of species’ assessment and be used to target conservation actions.

The IUCN species’ assessment process is the international standard for estimating extinction risk (Mace et al., 2008), offering estimates of past, current, and future declines in species (Rondinini et al., 2014). The current IUCN Red List guidelines (IUCN Standards and Petitions Committee, 2024) provide no explicit guidance related to the use of citizen science data in extinction risk assessment, which limits the capacity of citizen scientists to provide essential data.

Citizen science data collection is already helping address the shortfall in information for undertaking threat assessments for plants and animals in marine and terrestrial environments (Camins et al., 2024; Mandeville et al., 2023; Van der Colff et al., 2023), but the comprehensive and nuanced IUCN Red List criteria mean there may not be a straightforward entry point for existing or new citizen science data into the assessment process. The criteria require taxon‐specific data (see below “A QUICK GUIDE TO THE IUCN RED LIST CRITERIA FOR CITIZEN SCIENTISTS”) (IUCN Standards and Petitions Committee, 2024). Scientists working in extinction risk assessment may not be engaging at the planning phase, with practitioners designing new citizen science projects, leaving a gulf between the expectations and realities of how informative data might be. Steven et al. (2019) found a strong foundation for public participation in threatened species conservation activities but identified the need to integrate the principles of best practice citizen science into threatened species assessment. More recently, Fontaine et al. (2022) and Van der Colff et al. (2023) found that citizen science contributes to rare or threatened animal and plant monitoring and show that there is scope for this type of data to be applied in IUCN assessments more systematically. Similarly, although the assessments for red handfish (*Thymichthys politus*) (Stuart‐Smith et al., 2020) and tea‐tree fingers (*Hypocreopsis amplectens*) (May, 2021) were informed by data from the citizen science programs Reef Life Survey and Fungimap, respectively, this data integration is not yet routine. Making similar data use commonplace, rather than exceptional, can boost capacity to assess and protect more species.

There is a tremendous wealth of existing data collected through citizen science platforms, such as iNaturalist (https://www.inaturalist.org) and eBird (https://ebird.org), that both house hundreds of millions of biodiversity records across a wide range of taxa and regions. These platforms demonstrate the strong potential for large‐scale, multitaxa data collection that can be directly applied to conservation assessments, including IUCN assessments, especially when records are verified by specialists (Gardiner & Bachman, 2016; Samain et al., 2023). Leveraging these platforms and integrating their data sets with professionally collected data could expedite data gathering, increase geographic and taxonomic coverage, and provide important insights into species’ current status, distribution, and trends (Camins et al., 2024). However, despite the vast potential of citizen science data, they remain underutilized in conservation assessments and actions.

We explored the benefits of existing and new citizen science programs to extinction risk assessments when data collection efforts are tailored to the requirements of the five specific IUCN Red List criteria: data on population size reduction (criterion A); geographic range (criterion B); small population size and decline (criterion C); very small or restricted population (criterion D); and quantitative analysis (criterion E). We categorized citizen science outputs into five broad types (i.e., occurrence data, presence–absence observations, structured surveys, physical samples, and narratives) and examined them relative to each IUCN Red List criteria to consider their suitability for assessing species. We considered case studies of two citizen science programs that have delivered useful data for IUCN assessments: FrogID (Rowley et al., 2019; https://www.frogid.net.au/) and Fungimap (https://fungimap.org.au/). Based on our findings, we devised guidance on caveats, data integration, and designing programs that simultaneously maximize citizen scientist participation and benefits to IUCN's red‐listing process.

## FIVE TYPES OF CITIZEN SCIENCE OUTPUTS FOR IUCN ASSESSMENTS

Citizen science encompasses a range of different outputs and data types, and platforms for data collection can target many simultaneously. Although we recognize that citizen science data types have been grouped and presented in a variety of ways (e.g., Pocock et al., 2018; Stevenson et al., 2021), we categorized citizen science into five data outputs so we could map them against their utility in meeting IUCN criteria.

### Occurrence data

To date, the most significant contribution (in terms of volume) of citizen science to conservation science has been primary data collection (Bonney et al., 2016); occurrence data are the most common form of data collected (i.e., species records that typically include spatial coordinates and temporal information [Sun et al., 2021]). In addition to spatial information, observations also tend to be accompanied by vouchers (usually an image or sound recording) (Mesaglio, 2024; Sun et al., 2021), although there are exceptions to this, such as eBird, for which no vouchers are required. The platform iNaturalist has collated over 180 million biodiversity occurrences, most with images, and other initiatives, such as iSeahorse (https://www.inaturalist.org/projects/iseahorse), whose thousands of seahorse records have already contributed to IUCN assessments and updates, provide an invaluable model for effective use of this data type (Camins et al., 2024). Occurrence data from citizen scientists will continue to grow, but they require validation and careful preparation to be used in IUCN assessments (May, 2021; Mueller et al., 2022). Validation will ideally be via Red List authorities managed by the IUCN, although this task will be large and will require substantial additional resources.

In line with other forms of biodiversity data, the quality of occurrence data from citizen scientists is improving rapidly, in part due to the integration of approaches such as machine learning to suggest species identifications alongside expert validation (Campbell et al., 2023; Ceccaroni et al., 2019; McClure et al., 2020). As a result, point observations from citizen science platforms are now a highly valuable source of spatiotemporal information. Where the number of occurrences of a target group of species is high, it may also be possible to infer absence (i.e., pseudoabsence) from occurrence data (Stokland et al., 2011). This type of inference can be useful in IUCN assessments but requires high sampling density to ensure accuracy.

### Presence–absence observations

Presence–absence observations provide evidence that a species has not been detected at a location—an important extension of occurrence data. Knowing that a species has not been detected from a site despite extensive surveying is highly informative for a range of ecological questions (Feldman et al., 2021). Currently, absence observations are not routinely targeted for collection in most citizen science projects (Aceves‐Bueno et al., 2017). This may be because there are inherent risks involved in declaring a species absent from a location related to factors such as search effort, observer skill in detecting the species, and detectability of the organism (MacKenzie, 2005). Gathering absence observations may, in some instances, require citizen scientists to engage more deeply in the basic principles of biological survey, which in turn requires more dedication than simply uploading images with geolocations as with occurrence data.

Absences (or rather, detectability or site occupancy) can be imputed from presence‐only observations with mathematical approaches for analyzing large unstructured data sets that account for survey effort (Callaghan, Bowler, et al., 2022; Sicacha‐Parada et al., 2021). Methods also exist for estimating occupancy based on repeated (MacKenzie et al., 2002) or single visits (Lele et al., 2012) or time to detection (Henry et al., 2020). The common thread among these methods, however, is that scientists must know that a survey was performed; how long the survey was carried out for; and what the surveyor was looking for.

### Structured surveys

These are outputs that stem from the participant answering a series of structured questions about the target organism and its environment (Kelling et al., 2019), often over a defined period or at a specific location (Callaghan et al., 2018). This may include multiple tasks, such as counting individuals (abundance), estimating the age class of the individuals present, or recording environmental conditions or the presence of threats at an occupied or unoccupied site. For example, when adding observations to the iNaturalist project redlist (s Afr) (https://www.inaturalist.org/projects/redlist‐s‐afr), designed to assist evaluations of southern African species, users must complete four basic “observation fields.” These fields augment the basic occurrence data and provide valuable additional information for assessments.

Structured surveys may also target the gathering of vital metadata that indicate the observer's skills and techniques used, enriching the scientific value of the observations (Kelling et al., 2019). Using structured surveys can help address spatial and temporal biases in citizen science data that arise from the greater collection of data for species that are more obvious or easy to identify and occur in accessible areas or under favorable conditions (e.g., spring) (Callaghan et al., 2018). However, effective structured surveys typically require sustained engagement and a high level of planning, development, and training of citizen scientists, which may limit the volume of data collected.

Clear protocols for assessing the magnitude and impact of threats will assist observers to contribute nuanced data (Altwegg & Nichols, 2019). In some cases, threat information could also be retrospectively added to citizen science observations through spatial analysis, such as automated image analysis to estimate cover (Williams et al., 2019). To do this well, however, protocols and training would need to be vastly scaled up to help citizen scientists standardize their measurements.

Some citizen science projects facilitate the collection of structured survey data (e.g., redlist (s Afr) [https://www.inaturalist.org/projects/redlist‐s‐afr] and Flora Connections in Australia [https://inaturalist.org/projects/flora‐connections]). Within these projects, guidelines, data sheets, or both have been prepared to enable citizen scientists to record data in a systematic way and include variables, such as habitat condition and threats, that are important for assessments. However, these kinds of projects rely on dedicated participants and may therefore be difficult to scale up (Kelling et al., 2019). Exceptions exist, however, such as very successful initiatives focused on documenting roadkill and dead tree monitoring (Heigl et al., 2022; Nolan et al., 2020).

### Physical samples

Citizen scientists can be trained in the ethical collection of physical samples associated with a species presence (or absence) at a location, such as scat, or via environmental samples (e.g., soil or water) (Fraisl et al., 2022). Physical samples typically require laboratory analysis to confirm a presence or elucidate environmental information, such as pollutants. Samples may accompany one or more of the four other citizen science outputs and typically require significant additional funding to collect, transport, analyze, and interpret. Permitting is typically required for sample collection, and can be complicated to implement. Despite this, several projects successfully collect citizen science physical samples. In the Sudden Oak Death project, for example, samples are collected by the public and sent for laboratory analysis to confirm the presence of the causal agent *Phytophthora ramorum*, which has caused the death of thousands of native oak and tanoak trees (*Lithocarpus densiflorus*) (Meentemeyer et al., 2015). A similar approach is taken by Echidna CSI, where citizen scientists collect scat samples (Perry et al., 2022). To overcome barriers to facilitating and processing sample collections, we recommend that projects be developed and implemented in stages that incrementally increase submissions as pipelines for sample collection and analysis are refined to reliably process large volumes.

### Narratives

Narratives often take the form of collected stories or oral histories. Subjects give prior and informed consent, and their memories and conversations are recorded and transcribed (Hecker et al., 2018). Narratives also include cultural expressions, such as art, song, and dance, and can play an important role in bridging the gap between science and society (Campbell et al., 2022; Ens et al., 2016). Citizen science initiatives may not yet cater to the vast array of communication styles and knowledge sharing protocols that exist (Richter et al., 2019), particularly those within Indigenous communities (Ens et al., 2016), but this is likely to change. By integrating storytelling methods into citizen science approaches, the scope and reach of projects can be expanded to capture previously underrepresented groups (Veeckman et al., 2023). Where species information is targeted, stories may yield data on distributions (presence or absence), changes in abundance through time, and the threats that contribute to declines (Russell et al., 2023). Narratives are likely to yield data that are primarily qualitative and sometimes highly anecdotal, meaning careful collection, qualitative analysis, interpretation, and application are required to enable their use in IUCN assessments (IUCN, 2024).

In 2021, the IUCN produced a white paper that examined the issue of inclusion of Indigenous and local knowledge (ILK) in species’ assessments (IUCN, 2022). This work recognizes the value and importance of local and Indigenous knowledge systems in supporting extinction risk assessment and provides practical guidance about how to move forward. Further calls for combining local and Indigenous knowledge with other scientific understandings through citizen science are gaining attention (Tengö et al., 2021), and local ecological knowledge is increasingly being integrated into decision‐making. For instance, Prober et al. (2019) used targeted elicitation of local ecological knowledge to understand climate change impacts on species and to integrate this into planning and decision‐making. Indigenous‐led citizen or community science programs have the capacity to reach remote, understudied regions where Indigenous Peoples maintain close connection to country including unique spatial and temporal knowledge of species. Past observations or memory knowledge of species distributions, ecology, and threats held by communities, including Indigenous knowledge custodians, have filled significant species data gaps in science databases (Prober et al., 2019; Russell et al., 2023; Skroblin et al., 2021; Ziembicki et al., 2013).

There are, however, several challenges to overcome in the design, implementation, and interpretation of projects that document cultural knowledge of species (Goolmeer & van Leeuwen, 2023). Importantly, when combining Indigenous knowledge with ecological data, the Indigenous data governance C.A.R.E. principles (collective benefit, authority to control, responsibility, ethics) (Jennings et al., 2023) and mutual benefits must be respected and incorporated. Logistical difficulties, such as access, internet coverage, cost, and field work in isolated or remote regions, also need to be considered when seeking to record narratives of Indigenous People on their lands. If conducted respectfully and with a view for mutual benefits, making better use of narratives can support conservation of species and equally threatened Indigenous knowledge, cultures, and language (Tengö et al., 2021). Similar to occurrence data, improved collaboration with communities may embed narratives in IUCN assessments. Going forward, we recommend investment in quantitative analyses to better understand how much Indigenous knowledge data are presently incorporated into conservation assessments.

## A QUICK GUIDE TO THE IUCN RED LIST CRITERIA FOR CITIZEN SCIENTISTS

The IUCN's approach to species extinction risk assessment is built around five criteria (A–E). Short explanations of the criteria and their application are provided below (and in detail at https://www.iucnredlist.org/resources/categories‐and‐criteria). Each criterion targets a specific aspect of species biology, demography, or ecology, which can be subject to decline. Species are assessed as being in one of seven risk categories (LC, least concern; NT, near threatened; VU, vulnerable; EN, endangered; CR, critically endangered; EW, extinct in the wild; EX, extinct). At least one criterion must be met to list species as threatened (i.e., VU, EN, or CR). Species can also be classified as data deficient (DD), which means information for assessment is insufficient to distinguish between a threatened category and LC. Table 1 shows our assessment of the utility of each data type in assessment. Some IUCN assessment terms, such as *location*, *population*, and *severe fragmentation*, have nuanced definitions when applied to the criteria. Because the meaning of these terms may not be intuitive, and differ from general understanding, Table 2 offers basic guidance on how to interpret what they target. Full definitions and explanations are available in the IUCN Red List Guidelines (IUCN, 2024).

### Population size reduction (criterion A)

Assessing population size requires data on the magnitude of reduction in population size or indicators in population size (e.g., area of occupancy [AOO], extent of occurrence [EOO]) over the longer of 10 years or 3 generations coupled with information on prevalence of the threats that have precipitated the reduction.

### Geographic range (criterion B)

Geographic range is used to assess species based on knowledge of geographic range; severe fragmentation; the number of threat‐defined locations; observations of continuing decline; and fluctuation in occurrence or occupancy. Range is assessed via EOO (total area of a minimum convex polygon encompassing all occurrences of the species) and AOO (total area encompassed by the number of unique 2 × 2‐km grid cells occupied).

### Small population size and decline (criterion C)

Criterion C differs from criterion A because it emphasizes both small population size and decline. An indication of the total number of mature individuals is required as is an understanding of generation length (i.e., the average age of parents in the individuals present) and continuing decline quantified through individual subpopulation counts or based on fluctuations in the number of mature individuals.

### Very small or restricted population (criterion D)

This criterion is similar to criterion C but focused specifically on identifying species with very small or restricted numbers of mature individuals.

### Quantitative analysis (criterion E)

Quantitative analysis is typically conducted as a population viability analysis that quantifies the probability of extinction in the wild. The probability is based on the percent decline of individuals over the shorter of 10 years or 3 generations. This is the most data hungry of the IUCN Red List criteria (Kindvall & Gärdenfors, 2003).

**TABLE 1:** Citizen science outputs and their potential utility in International Union for Conservation of Nature (IUCN) species’ assessments by criteria and subcriteria.

				Applicable IUCN Red List criteria and subcriteria				
				A	B	C	D	E
Citizen science output	Definitions	Benefits	Considerations	Population size reduction	Geographic range	Small population size and decline	Very small or restricted population	Quantitative analysis
Occurrence data	One‐off record of the presence of an individual at a location. Includes latitude and longitude coordinates, time and date stamp, and typically a photograph; may also be derived from remote sampling methods (e.g., acoustics, cameras) or from trace sampling (e.g., eDNA).	Abundant and easy to acquire; takes advantage of near ubiquitous technology (i.e., mobile phones); requires no specialized skills to gather; already collected in vast quantities by citizen scientists	Provide informative data for mapping species distributions Where observation effort occurs evenly through time, distributional change for a species may be detected Acoustic recordings and eDNA that lead to basic observations require significant infrastructure to collect, store, and process and require verification from other survey methods (see “Structured surveys” below) Recording precision of an observation is important; occurrences with a high degree of geographic uncertainty may need to be discarded so as to not create misleading extent of occupancy (EOO) or area of occupancy (AOO) values	May be useful for demonstrating a decline in indicators of population size (EOO and AOO) if analyzed as a time series (i.e., species now consistently occupies a reduced number of sites, and there has been consistently high sampling over time [i.e., inferred absence])	Useful for defining EOO and AOO under subcriteria B1 and B2 if the species identification is valid May be useful for demonstrating a decline in EOO and AOO if analyzed as a time series (i.e., the species now consistently occupies a reduced number of sites)	May be useful in very specific cases where the absence of occurrence records in a region well sampled by citizen scientists indicates a very small population size Generally, occurrence data will not indicate population size or decline	Useful for defining AOO under subcriteria D2 if the species identification is valid	No
Presence–absence observations	One‐off record of the presence or absence of an individual at a location following some level of survey effort; includes latitude and longitude coordinates, time and date stamp, and typically a photograph if present; may be derived from remote sampling methods (e.g., acoustics, cameras) or trace sampling (e.g., eDNA)	As for occurrence data but requires some specialist skills in survey methods and an ability to understand survey effort Can offer opportunities for science extension activities, such as training of an existing workforce or deep engagement with citizens keen to improve their skills base	Detecting true absence requires significant expertise and effort for most species and is rarely achieved even when trained specialists are used Failure to detect the species at a site without structured survey may not provide sufficient evidence absence Requires additional training of citizen scientists in basic survey methods	May be useful for demonstrating a decline in indicators of population size (EOO and AOO) if analyzed as a time series (i.e., species is now consistently absent from previously occupied sites)	Useful for defining EOO and AOO under subcriteria B1; B2 if the species identification is valid May be useful for demonstrating a decline in EOO and AOO if analyzed as a time series (i.e., species is now consistently not detected through time in previously occupied sites)	May be useful in very specific cases where the absence of occurrence records in a region well‐sampled by citizen scientists indicates a very small population size Generally, occurrence data will not indicate population size or decline	May be useful for demonstrating that species population is very restricted under subcriterion D2	No
Structured surveys	Systematic survey of a location guided by an expert‐designed survey and template for recording data Includes deliberate effort to target data collection for the skill level of participating citizen scientists, Data routinely collected using field apps	Can deliver targeted data for specific questions when carefully designed, including information on the presence and impact of threats Increases capacity for citizen scientists to contribute to science Can create greater longevity in engagement of citizen scientists in conservation projects if communication with participants is well maintained	Requires substantial prior planning to devise an appropriate survey structure relative to the aims of the project Design process should include experts familiar with appropriate methods of sampling for the species being targeted Surveys must be designed to avoid fatigue in citizen scientist by matching neatly with their likely skills	Useful for measuring population change under subcriterion A3b–e, if the survey is conducted for 10 years or 3 generations, or under A4a–e, if repeat surveys are conducted after establishing a baseline	Useful for tracking threat defined locations (B1a, B2a), continuing decline (B1b[i–v], B2b[i–v]), and extreme fluctuations (c[i–v]) where repeat surveys are conducted after establishing a baseline	Useful for detecting number of mature individuals and their continuing decline (C1; C2a[i, ii], b) or extreme fluctuations (C2b) where repeat surveys are conducted after establishing a baseline	Useful for counting number of mature individuals (D) or information on future threats, number of threat‐defined locations, and AOO under D2	Can provide data to conduct population viability analysis (PVA) or similar quantitative assessment
Physical samples	Samples associated with a species presence or absence at a location (e.g., scats or soil/water samples) May include sampling to detect presence at some point in time	Physical samples usually require laboratory analysis after collection to confirm a presence or elucidate information Samples work in conjunction with other outputs, such as presence–absence observations	Citizen scientists may require a higher level of sustained engagement for physical sampling to be successfully carried out Although they collect samples, they may not be involved directly in the scientific work, and communication with the scientist is necessary	Potentially useful for estimating population genetic structure and demography (A2–4a) and threats that leave genetic signatures (e.g., pathogens) (A2–4b–e)	Useful for estimating severe fragmentation through analysis of population genetic structure (B1a, B2a), presence of threats (e.g., pathogens) that can define a location (B1a, B2a), and detecting extreme fluctuations (B1c, B2c)	Potentially useful for estimating age of individuals or generation length in a population with genetic tools Useful for detecting extreme fluctuations (C2b)	Potentially useful for estimating age of individuals or generation length in a population with genetic tools for estimating number of mature individuals	Can provide data to conduct PVA or similar quantitative assessment
Narratives	Knowledge captured through mutually agreed terms May include narratives of local change in the environment from landholders or oral histories and stories from Indigenous People May be cross‐cultural	Can respectfully capture oral history information that may not be a good fit with established scientific methods Can include observations of past encounters with a species at a site that may be used to infer changes in distribution or threats present	Ethics of data capture and reuse need to be carefully negotiated to avoid exploitation and culturally inappropriate disclosures Requires highly skilled communicators and long‐term investment in establishing trusted relationships with communities	Potentially useful for documenting trends in species under A1, A2, A3, A4 (a–e)	Useful for defining EOO and AOO under subcriteria B1 and B2 Potentially useful for defining the number of threat‐defined locations (B1a, B2a; see Table 1) and for elements of B1b and B2b	Potentially useful for qualitative estimates of population size in the absence of numerical data and elements of generation length, such as time to maturity	Potentially useful for qualitative estimates of population size where mature individuals can be readily distinguished	Not likely to provide quantitative data for PVA

**TABLE 2:** Terms with nuanced meanings used in the International Union for Conservation of Nature (IUCN) Red List criteria.

IUCN term	IUCN definition (IUCN Standards and Petitions Committee, 2024)	Guidance on use
Location	“A geographically or ecologically distinct area in which a single threatening event can rapidly affect all individuals of the taxon present.”	A location is a threat‐based area and may encompass multiple occurrences of a species that can be simultaneously threatened by single event. It is not therefore equivalent to the more general understanding of the term location, which is a place or position in the landscape.
Population	“The total number of individuals of the taxon.”	A population refers to all of the individuals of the species present throughout its range. This term cannot be interchanged with population size, which refers to mature individuals only in the Red List criteria.
Population size	“…measured as numbers of mature individuals only.”	Counts (abundance) of the number of mature individuals across all areas occupied by the species.
Mature individuals	“The number of individuals known, estimated or inferred to be capable of reproduction.”	Factors such as the abundance and density of individuals, presence of sex ratio bias, fluctuations in population size, clonality, timing of survey, and reproductive status of reintroduced individuals must be considered in estimates.
Subpopulation	“…geographically or otherwise distinct groups in the population between which there is little demographic or genetic exchange (typically one successful migrant individual or gamete per year or less).”	Subpopulations are significant units for applying the RedList criteria, which are used to assess the additional risks to a species when its population (as defined above) is divided into smaller units or the majority of individuals occur in only one unit. Subpopulations have high, but not complete, genetic and demographic separation.
Extreme fluctuation	“…occur…where population size or distribution area varies widely, rapidly and frequently, typically with a variation greater than one order of magnitude (i.e., a tenfold increase or decrease).”	This definition recognizes that high variability in population growth rates increases risk of extinction. The entire population, as defined above, should fluctuate by at least an order of magnitude to be considered extreme, and all subpopulations must be connected by dispersal with synchronized fluctuations. Fluctuations in totally isolated subpopulations can also be considered extreme where all are changing in size by an order of magnitude.
Severe fragmentation	“…most…individuals are found in small and relatively isolated subpopulations.”	Differs from the concept of habitat fragmentation, which is commonly used in conservation science, although habitat fragmentation can be used to infer severe fragmentation of the population if species‐specific knowledge of factors, such as dispersal distance and densities, in subpopulations is available. Spatial data can be used to infer fragmentation in a species, when “most (>50%) of its total area of occupancy is in habitat patches that are (1) smaller than would be required to support a viable population, and (2) separated from other habitat patches by a large distance relative to dispersal kernel of the species.”
Generation length	“…the average age of parents of the current cohort (i.e., newborn individuals in the population). Generation length therefore reflects the turnover rate of breeding individuals in a population. Generation length is greater than the age at first breeding and less than the age of the oldest breeding individual, except in taxa that breed only once.”	Indicates the rate of turnover in breeding individuals and therefore how fast a population can respond to threats and declines. This parameter is used throughout the Red List to calibrate time‐based estimates of decline specific to the taxon being assessed. Several methods for estimating generation length are provided in the IUCN Guidelines (IUCN Standards and Petitions Committee, 2024); a method appropriate to the biology of the taxon being assessed should be chosen.

## CASE STUDIES OF CITIZEN SCIENCE PROJECTS DELIVERING USEFUL DATA FOR IUCN ASSESSMENTS

The following case studies illustrate how combining leadership from taxon specialists with the enthusiasm and skills of citizen scientists has facilitated IUCN assessments of amphibians and fungi.

### Using FrogID to inform extinction risk assessment

FrogID is a national citizen science project through which data on Australia's frog species are gathered (Rowley et al., 2020, 2019). The project uses a smartphone app that allows participants to submit audio recordings of calling frogs and associated metadata, including location, time, and date (i.e., occurrence data [Table 1]). The frogs calling in each submission are identified to species (where possible) by specialists. The project has gathered over 1,000,000 frog records in just over 6 years, and the data are being used in conservation assessments for several threatened frog species. For example, in 2023, the Sphagnum Frog (*Philoria sphagnicola*) was listed as VU under Australian legislation based on the IUCN‘s assessment, partly as a result of data from FrogID (Department of Environment, 2024a). This assessment followed the 2019–2020 Black Summer bushfires in Australia, and a mix of FrogID records from unburned and burned sites was used to infer decline under criterion B. Similarly, for the Davies’ tree frog (*Litoria daviesae*), presence was detected post fire at seven low to moderately burned sites. Although the population trend of this species requires further targeted surveying to confirm, the FrogID observations were sufficient to infer a possible population decline, including a potential loss of subpopulations. This resulted in a VU listing based on IUCN criterion B under Australian legislation (Department of Environment, 2024b), but this designation required specific expertise from within the FrogID project and familiarity with the IUCN Red List Criteria to analyze and interpret the data sets. Therefore, the need for collaboration among those preparing assessments and curators of citizen science projects remains key to listing outcomes. Also critical was the inclusion of FrogID data in government data sets used for decision‐making (many citizen science projects are not included due to a perceived lack of data quality).

### Fungimap and IUCN Fungal Red List extinction risk assessments

Fungimap Inc. is dedicated to improving knowledge and conservation of fungi in Australia. Initially, its focus was on recording target species from Fungimap's field guide (Grey & Grey, 2005). Target species were selected for identifiability and mostly broad distribution (May, 2021). Records of all species of fungi are accepted via the Fungimap Australia project (https://www.inaturalist.org/projects/fungimap‐australia). Fungimap has facilitated the collation of over 200,000 records with location and date and often information on habitat and substrate. These citizen science records, mainly from occurrence data (Table 1), were vital for threat assessments of 19 species of Australian fungi at a Global Fungal Red List Initiative workshop (https://redlist.info/en/iucn/welcome) in 2019 (https://www.australasianmycologicalsociety.com/conservation‐group). Assessments drew on mycologist and Fungimap citizen scientist records. Thirteen species were assessed as threatened under 3 of the 5 criteria, including B (B1 *Heimioporus australis*; B2 Fischer's egg [*Claustula fischeri*]), C (C1 *Austroboletus viscidoviridis*; C2 tea‐tree fingers), and D (D1 *Auriscalpium* sp. “Blackwood”]). The high background volume of Fungimap records from areas where threatened species occurred was useful in demonstrating that the paucity of records and small range of some species was genuine and not due to underrecording (e.g., see IUCN Red List assessments by Buchanan and May [2019]). Without this calibration with citizen science records, most species would have been data deficient.

## MAXIMIZING THE USE OF CITIZEN SCIENCE DATA IN IUCN RED LIST ASSESSMENTS AND CONSERVATION

Citizen science data are rapidly growing in scope and influence, often eclipsing the volume of data collected by specialists. For example, Roger et al. (2023) compared citizen‐derived versus noncitizen observation records from a sample of common and range‐restricted species. They found that citizen science data contribute most information on occurrences, including adding new locations and infilling occurrences in a species’ range. Indeed, a great benefit of citizen science data is when data sets are integrated to create big data that can inform future sampling events, with data then used in threat assessments. Strategic national and global frameworks for spatial and taxonomic gap filling are urgently needed, with some models already emerging (e.g., for English Lepidoptera [Rolph et al., 2023]). Additionally, data aggregators must lower barriers to the integration of citizen science data sets into biodiversity portals, thereby increasing accessibility and visibility of the contributions of citizen scientists to biodiversity knowledge. We emphasize the importance of greater engagement by specialists with citizen science platforms, such as iNaturalist, to increase the number of high‐quality, specialist‐verified records that may then be used in IUCN assessments (Callaghan, Mesaglio, et al., 2022), including in the examination of taxa currently considered DD (Cazalis et al., 2023). We also suggest that, where relevant, citizen scientists seek out training opportunities around IUCN assessments that can increase their understanding of the process.

## CAVEATS TO THE USE OF CITIZEN SCIENCE DATA FOR THREATENED SPECIES

Part of the reluctance to fully engage the citizen science community with threatened species conservation likely relates to sensitivities around sharing of data for taxa at risk of extinction (Soroye et al., 2022; Tiago, 2016; Tulloch et al., 2018). Citizen science brings many potential opportunities to extinction assessment, yet the democratization of science is also perceived to heighten risk. The arguments for and against actively managing these risks have been outlined yet remain unresolved (Chapman, 2020). Yet, the consequences of not sharing data about species under assessment may be dire. Chapman (2020) found that restricting species data can place them in inadvertent danger by withholding knowledge of locations subject to development (Lindenmayer & Scheele, 2017). The simplest and most common response is to not share unmodified raw data on threatened species, even in citizen science. For example, iNaturalist and nodes of the Global Biodiversity Information Facility (GBIF), such as the Atlas of Living Australia and Canadian Biodiversity Information Facility, practice “taxon geoprivacy” by obscuring locations of sensitive species noted in official lists before displaying publicly or providing to third parties (Mesaglio, 2024; Roger et al., 2023). GBIF has developed a best practice approach to sensitive species data (Chapman, 2020), and standard methods for obscuration are in the process of being implemented in some places (e.g., National Framework for Sharing Restricted Access Data) (Atlas of Living Australia, 2023). Currently, data can enter large aggregators from more than one custodian and appear as multiple erroneous data points (Chapman, 2020). Ironically, this is exacerbated by another sensitive data response, the withholding of observer's personal details, which might enable two geographically orphaned obfuscated points from being recognized as the same point.

Taxonomic biases in biodiversity data, including citizen science, are well documented: vertebrates, especially birds, are overrepresented (Troudet et al., 2017) relative to groups such as invertebrates, plants, and fungi, which tend to be greatly underrepresented (Cornwell et al., 2019; Gallagher et al., 2023; Mesaglio & Callaghan, 2021). Bias also stems from higher representation of charismatic taxa and colorful and active species (Mesaglio et al., 2023; Ward, 2014). For many taxonomic groups, photographs can be insufficient to confirm a species identity, for example, when key taxonomic characters require microscopy or dissection, compounding issues of taxonomic bias. A pragmatic solution may be to focus extinction risk assessment on the most charismatic and easily identifiable taxa (target species) in underrepresented groups, for which citizen science can collect high‐quality data (Mesaglio et al., 2023).

Bias can also be geographic and temporal. Many citizen science data sets are heavily biased toward roads and easily accessed sites, a pattern consistent across many taxa and locations (Botts et al., 2011; Petrovan et al., 2020). Similarly, large population centers and frequently visited sites tend to be much sampled (Geldmann et al., 2016; Mair & Ruete, 2016) compared with sparsely populated areas (Mesaglio & Callaghan, 2021). These forms of bias are not unique to citizen science data—they also emerge in specialist‐derived data sets (Binley & Bennett, 2023; Botts et al., 2011; Daru et al., 2018). Citizen science may amplify existing biases, however, and conservation assessments that use this information must be careful to account for this issue (García‐Roselló et al., 2023). Given the voluntary nature of citizen science data, spatial and temporal biases may be difficult to overcome but can be complemented by targeted professional surveys in difficult‐to‐access areas and periods. Also, citizen science can be used to actively address biases in professional data by providing data from private property (Callaghan et al., 2021) or areas that may be inaccessible after disasters (Kirchhoff et al., 2021; Roger & Kinsela, 2023).

## CONCLUDING REMARKS

Our aim was to highlight the growth of citizen science in contributing effective data to threatened species assessments and how to continue to support this effort with careful guidance and program design. Although citizen science is unquestionably already contributing to conservation science, simply encouraging more ad hoc occurrence records may not be sufficient to inform threatened species listing, possibly resulting in unmet promises and expectations for citizen scientists. Creating clearer links between the IUCN assessment process and citizen science programs may deliver more targeted information about extinction risk. We therefore advocate for using citizen science data to inform IUCN assessments through planning targeted projects from the outset, more comprehensive efforts, such as sample collection or structured surveys, or as a complementary data stream integrated with professional data.

The IUCN assessments may also need to evolve to accommodate alternative sources of knowledge. Although the rigor of the IUCN Red List criteria may deter some assessors from using citizen science data, the guidelines for assessment (IUCN, 2024) explicitly state that projections, assumptions, and inferences are justified, and we argue this could extend to the use of citizen science data. Assessment methods must also keep pace with the rapid emergence of citizen science data as a major source of information on biodiversity globally and the advent of both quantitative techniques (Schultz et al., 2017) and artificial intelligence (Ceccaroni et al., 2019). For example, there is promising work emerging from the development of dynamic range maps derived (in part) from citizen science observations (Huang et al., 2021; Oliver et al., 2024). Population size estimates may also be possible through secondary analyses of vouchers, such as disease assessment, life stage, or reproductive success. Finally, population trend analysis may also be possible via analysis of mortality. We call for greater integration of the tremendous amounts of existing citizen science data with professionally collected data, for stronger efforts to implement monitoring schemes that provide species abundance data, and for globally harmonized metrics to provide a more accurate quantification of changing biodiversity patterns (Fraixedas et al., 2022). Ultimately, citizen science remains one of the best mechanisms for communities to contribute to biodiversity conservation and will only become more crucial in the face of continuing biodiversity loss.
